# Effect of the Degree of Polymerization of Fructans on Ex Vivo Fermented Human Gut Microbiome

**DOI:** 10.3390/nu11061293

**Published:** 2019-06-07

**Authors:** Erola Astó, Iago Méndez, Maria Rodríguez-Prado, Jordi Cuñé, Jordi Espadaler, Andreu Farran-Codina

**Affiliations:** 1 AB-Biotics, S.A, ESADE Creapolis, Av. Torre Blanca, 57, E-08172 Sant Cugat del Vallès (Barcelona), Spain; asto@ab-biotics.com (E.A.); mendez@ab-biotics.com (I.M.); jordicune@gmail.com (J.C.); 2 Department of Nutrition, Food Science, and Gastronomy, XaRTA—INSA, Faculty of Pharmacy, University of Barcelona, Campus de l’Alimentació de Torribera, Av. Prat de la Riba, 171, E-08921 Santa Coloma de Gramenet, Spain; afarran@ub.edu; 3 Animal Nutrition and Welfare Service (SNIBA), Building V. Office V0-308, Autonomous University of Barcelona, C/ Travessera dels Turons s/n, E-08193 Bellaterra (Barcelona), Spain; maria.rodriguez.prado@uab.cat

**Keywords:** prebiotic, inulin-type fructan, fructooligosaccharide, polymerization degree, metagenomics, microbiome

## Abstract

Prebiotic supplements are used to promote gastrointestinal health by stimulating beneficial bacteria. The aim of this study was to compare the potential prebiotic effects of fructans with increasing degrees of polymerization, namely fructooligosaccharides (FOS) and inulins with a low and high polymerization degree (LPDI and HPDI, respectively), using an ex vivo fermentation system to simulate the colonic environment. The system was inoculated with pooled feces from three healthy donors with the same baseline enterotype. Changes in microbiota composition were measured by 16S metagenomic sequencing after 2, 7, and 14 days of fermentation, and acid production was measured throughout the experiment. Alpha-diversity decreased upon inoculation of the ex vivo fermentation under all treatments. Composition changed significantly across both treatments and time (ANOSIM *p* < 0.005 for both factors). HPDI and LPDI seemed to be similar to each other regarding composition and acidification activity, but different from the control and FOS. FOS differed from the control in terms of composition but not acidification. HDPI restored alpha-diversity on day 14 as compared to the control (Bonferroni *p* < 0.05). In conclusion, the prebiotic activity of fructans appears to depend on the degree of polymerization, with LPDI and especially HPDI having a greater effect than FOS.

## 1. Introduction

It is generally accepted that the bacterial community in the human gastrointestinal tract has a great impact on intestinal functionality and human health.The colon is the most colonized region within the gastrointestinal tract, where 10^11^–10^12^ cells/mL have been detected [1,2]. The microbes in our bodies have been estimated to encode 100-fold more unique genes than our own genome [3,4]. High-quality data from the US Human Microbiome Project (HMP) [5], European Metagenomics of the Human Intestinal Tract (MetaHIT) [6] and several other studies have now demonstrated the beneficial role of the normal gut microbiota in health down to the genetic level [6,7]. Most bacterial species colonizing the human gastrointestinal tract belong to the phyla Firmicutes and Bacteroidetes, while species of the phyla Actinobacteria, Proteobacteria, and Verrucomicrobia exist in lower numbers. The number of bacterial species within the human intestinal microbiota has often been estimated to be in the range of 500 to over 1000 species [8]. In a healthy state, they contribute nutrients and energy to the host via the fermentation of nondigestible dietary components in the large intestine, and a balance is maintained with the host’s metabolism and immune system [9,10].

The prebiotic concept was first defined in 1995 as a “non-digestible food ingredient that beneficially affects the host by selectively stimulating the growth and/or activity of one or a limited number of bacteria already resident in the colon” [11]. The International Scientific Association for Probiotics and Prebiotics (ISAPP) recently published a consensus statement to update the definition and scope of prebiotics. The statement defines a prebiotic as “a substrate that is selectively utilized by host microorganisms conferring a health benefit” [12]. The scope of this updated definition is therefore wider, as it implies that the modulatory effect of prebiotics is not necessarily limited to gastrointestinal microbiota (i.e., it can be administered to microbiota-colonized sites other than the gut). Furthermore, the ISAPP consensus statement emphasizes that the definition of prebiotics should not be limited to carbohydrate-based compounds, but should apply to any compound that can be used by the microbiota to confer a health benefit. Some examples are polyphenols and polyunsaturated fatty acids. The document also addresses the difficulties involved in providing definitive proof of causality when assessing the beneficial effects of a prebiotic. Still, it states that the health benefits of a prebiotic must be confirmed in the target host for its intended use and should always be mediated by the microbiota.

Inulin-type fructans (ITF) are the best-documented oligosaccharides for their effect on intestinal *Bifidobacteria* and are considered to be important prebiotic substrates [13]. Fructans can also be described by their polymerization degree (PD). Inulin-type fructans consisting of DP ≥10 units are considered long chain, while inulin-type fructans with a DP of <10 are considered short chain [14]. Nutritional studies have recommended a combination of inulins with different chain lengths in order to maximize their fermentative and prebiotic effects [13]. Beneficial effects of inulin and/or oligofructose on the intestinal microbiota, have been demonstrated in adults, such as increasing *Bifidobacteria* and *Lactobacilli*, as well as increasing butyrate production [15,16,17]. Moreover, the prebiotic activity of inulin and oligofructose seems to translate into beneficial health effects such as improved glycemic control, reduced plasma triglycerides, improved barrier function, and decreased oxidative stress [14]. However, not all fructans seem to produce the same effects on the gut microbiota [18,19,20].

Ex vivo fermentation models are considered reliable tools to assess how microbial populations are altered by gastrointestinal environments such as prebiotics [21]. The common purpose of ex vivo gut fermentation models is to cultivate a complex intestinal microbiota under controlled environmental conditions with the aim of carrying out microbial modulation and metabolism studies. Although there are several well-studied and validated complex in vitro models of human microbiota (e.g., the Simulator of the Human Intestinal Microbial Ecosystem (SHIME) [22,23], the TNO Gastrointestinal Model (TIM-2) [24] and the Three-Stage Compound Continuous Culture System [25], we were interested in performing a simpler continuous culture system with one single stage.

Several studies have classified the human intestinal microbiota into three enterotypes in order to reduce the complexity and facilitate the identification of microbiota compositional patterns [26,27]: enterotype 1, which has *Bacteroides* as its best indicator; enterotype 2, which is driven by *Prevotella*, a genus whose abundance is inversely correlated with *Bacteroides*; and enterotype 3, which is distinguished by a significant presence of Firmicutes especially of the *Ruminococcus* genus [26,27,28]. However, this classification should be treated with caution, as some authors have suggested that microbiota could be divided into two enterotypes, where *Bacteroides* enterotype was fused with the less well distinguished Firmicutes enterotype while other authors have proposed four enterotypes, two of theme resembling previously the described *Bacteroides* and *Prevotella* enterotypes, while the other two are a more complex mixture with lower levels of *Bacteroides*, one dominated by a variety of populations affiliated within the Firmicutes phylum and the other combining higher levels of *Faecalibacterium* and *Ruminococcaceae* (both belonging to the Firmicutes phylum) with *Alistipes* (which belongs to the Bacteroidetes phylum) [28,29].

The objective of the study was to evaluate how the polymerization degree influences the prebiotic effect of various fructans (FOS and two inulins with differing degrees of polymerization) in an ex vivo fermented human microbiome, under conditions that simulated the transverse colon, taken from the Macfarlane model [25,30].

## 2. Materials and Methods 

### 2.1. Fructans Analyzed

Three commercial fructans with differing average fructosyl chain lengths (i.e., degree of polymerization) were chosen for this study.

1. Oligofructose (FOS): **Orafti^®^ P95** (BENEO-Orafti S.A., Tienen, Belgium), a short-chain inulin obtained through partial enzymatic hydrolysis and consisting of fructose units joined by β(2-1) linkages, with an end-standing glucose unit and an average polymerization degree of two to eight units.
2. Low-polymerization-degree inulin (LPDI): **Orafti^®^ GR** (BENEO-Orafti S.A., Tienen, Belgium), a granulated powder extracted primarily from chicory and consisting of fructose units joined by β(2-1) linkages, with an end-standing glucose unit and an average polymerization degree of ≥10.
3. High-polymerization-degree inulin (HPDI): **Orafti^®^ HPX** (BENEO-Orafti S.A., Tienen, Belgium), a high-performance inulin derived from chicory and consisting of fructose units joined by β(2-1) linkages, with an end-standing glucose unit and an average polymerization degree of ≥23.

The concentration of each prebiotic in each fermentation vessel was 12 g/L.

### 2.2. Donor Information

Inclusion criteria of volunteers were as follows: healthy young adults aged 18–30 years, a BMI of between 19 to 29.9 kg/m^2^, and type-3 or type-4 stools over the previous 72 h, as per the Bristol stool scale [31]. Exclusion criteria were as follows: infectious diseases in the three months prior to fecal donation, antibiotic consumption in the three months prior to donation, blood in the donation, and alcohol intake 72 h prior to donation. Participants were asked to fill out a 72 h dietary recall form and a food frequency questionnaire (Supplementary Table S1). Donors were required to consume a varied diet rich in fruit and vegetables (with a minimum of three portions of vegetables and two portions of fruit per day). 

All subjects gave their informed consent for inclusion before they participated in the study. The study was conducted in accordance with the Declaration of Helsinki, and the protocol was approved by the Ethics Committee of the University of Barcelona (IRB00003099).

### 2.3. Fecal Sample Collection and Inoculum Preparation

Fecal samples were collected (a minimum of 150 g from each donor) and kept in anaerobic conditions with Oxoid AnaeroGen (Thermo Scientific). The samples were homogenized by dissolution in glycerol/PBS (10% *w*/*v*) directly after collection to maximize preservation for the subsequent stages. The samples were frozen at −20 °C and stored in ready-to-use aliquots in the ex vivo fermentation [32,33]. Prior to fermentation, one aliquot from each donor was incubated for 1 h at 37 °C. 

### 2.4. Fermentation

Fermentation was carried out in a single-flow in vitro digestion system that simulated the transverse colon conditions defined previously [25,30]. The system consisted of eight independent units (500 mL amber glass jars), in which the fermentation conditions were monitored and controlled. The pH of the culture medium was maintained at 6.2 ± 0.2 by means of a pH meter (model PH 28, Crison Instruments S.A., Barcelona, Spain) connected to two peristaltic minipumps (8 RPM, 100 series pump; Williamson, Southwick, UK) to regulate the addition of HCl 2N or NaOH 3N. Temperature was monitored with a probe connected to a digital temperature controller (Autonics, Busan, South Korea) and maintained at 37 °C ± 0.5 with a circulating water bath (PolyScience, Niles, IL, USA). Anaerobic fermentation conditions were achieved by constant flushing of O_2_-free N_2_ gas. Liquid retention time was 48 h (2.083%/h dilution rate) by means of two precision peristaltic pumps, one for the culture medium input and one for the fermentation medium output (Masterflex Digital Console Drive; Cole-Parmer, Vernon Hills, IL, USA). All equipment and consumables used in the fermentation process were sterilized before the experiment and sampling started. 

The artificial colon simulator was fed a complex mixture of nutrients defined by Macfarlane and widely used in the literature [25,34,35]. This mixture was used as our basal medium, either supplemented with the fructans or alone as a control. The potentially interesting fructans were tested after being added to the Macfarlane composition, while the basal medium alone was used as a control treatment. An aliquot of 60 mL of the prepared fecal inoculum was added to each fermenter, together with 340 mL of the basal medium. The experiments were carried out in parallel in eight anaerobic jars (two replicas for each treatment with the basal medium or the basal medium plus fructans).

### 2.5. Metagenomics Analysis

#### 2.5.1. DNA Extraction

Bacterial DNA was extracted from 0.2 g of each sample using the PowerFecal™ DNA isolation kit (MO BIO, Carlsbad, CA, USA), in accordance with manufacturer’s instructions. DNA samples (100 μL) were stored at −20 °C until further processing. To assess for contamination from the laboratory or reagents, blank samples were processed at the same time. Samples obtained from the fermenters were processed for DNA extraction upon collection.

#### 2.5.2. PCR Amplification and Massive Sequencing

The V4 region of the 16S rRNA gene was amplified with the widely used primer pair F515 (5′- GTGYCAGCMGCCGCGGTAA-3′) and R806 (5′-GGACTACNVGGGTWTCTAAT-3′). Both primers included sequencing adaptors at the 5′ end and forward primers were tagged with different barcodes to pool samples in the same sequencing reaction. The PCR mixture (25 μL) contained 2 μL of DNA template (5 ng), 5 μL of 5× Phusion^®^ High-Fidelity Buffer, 2.5 μL of dNTPs (2 mM), 0.2 μM of each primer and 0.5 U of Phusion^®^ Hot Start II Taq Polymerase (Thermo Fisher, Waltham, MA, USA). 

The PCR thermal profile consisted of an initial denaturation at 98 °C for 30 s, followed by 30 cycles at 98 °C for 15 s, 55 °C for 15 s, 72 °C at 20 s and a final step at 72 °C for 7 min. To assess possible reagent contamination, each PCR reaction included a no template control (NTC) sample. For each amplicon, the quality and quantity were assessed with an Agilent 2100 Bioanalyzer and a Qubit^TM^ fluorometer, respectively. 

Each sequencing pool included 40 barcoded samples that were sequenced on an Ion Torrent Personal Genome Machine (PGM) with the Ion 318 Chip Kit v2 and the Ion PGM™ Sequencing 400 Kit (Life Technologies, Carlsbad, CA, USA), in accordance with manufacturer’s instructions. 

#### 2.5.3. Quality Control of the Sequences and OTU Picking

Raw sequencing reads were demultiplexed and quality filtered using QIIME 1.9.1 [36]. The reads included presented: a length greater than 225 bp; a mean quality score above 25 in a sliding window of 50 nucleotides; no mismatches on the primer; and default values for other quality parameters. The quality-filtered reads were subsequently processed with VSEARCH v1.1 pipeline [37]. An initial dereplication step was applied, then the reads were clustered into operational taxonomic units (OTUs) at 97% similarity with a de novo approach and, finally, de novo chimera checking was performed with the Uchime function. The raw OTU table was transferred into QIIME 1.9.1 and the taxonomic assignment of representative OTUs was performed using the Ribosomal Database Project (RDP) Classifier [38] against the Greengenes v13.8 database [39]. The sequences were aligned with PyNAST [40]. We sequentially applied some extra filtering steps in the aligned and taxonomy-assigned OTU table to filter out sequences belonging to chloroplasts and those belonging to the *Shewanellaceae* and *Halomonadaceae* families, which were represented in the NTC and considered as contamination due to the reagents.

#### 2.5.4. Data Analysis

Data obtained from relative abundance and reads for each sample were organized at the phylum, class, order, family, and genus levels. The alpha-diversity (within a given sample) was calculated at the genus level using two indexes: the exponential Shannon index [41] (i.e., the number of effective OTUs, or Hill number of order 1) and the inverse Simpson index [42] (i.e., the number of highly effective OTUs, or Hill number of order 2). Two-way analysis of variance (ANOVA) was used to assess the effect of treatment and time on alpha diversity, community stabilization (i.e., beta-diversity compared to the previous day) and acidification activity, with the Bonferroni post-test for pairwise analyses at each time point. The ANOVA was performed and the graphs generated with GraphPad Prism 5 (GraphPad Software Inc, San Diego, CA, USA). Beta-diversity (among samples, either from the same or a different treatment) was studied using two types of multivariate analysis at the genus level: non-metric multidimensional scaling (NMDS) for visual grouping, and analysis of similarities (ANOSIM) for statistical significance. More precisely, the significance of the effect of the treatment and time factors was evaluated with two-way ANOSIM, while pairwise differences between treatments on days 7–14 were evaluated with one-way ANOSIM. This procedure was performed with both the Sorensen [43] and Bray–Curtis [44] beta-diversity indexes, using the PAST 3.0 program [45]. 

## 3. Results

### 3.1. Relative Abundance

The samples from the three donors (two females and one male aged between 25 and 27) belonged to the Firmicutes-predominant enterotype according to the MetaHit classifier, and a 1:1:1 mixture was therefore prepared as an inoculum for all ex vivo experiments. 

**Figure 1:**
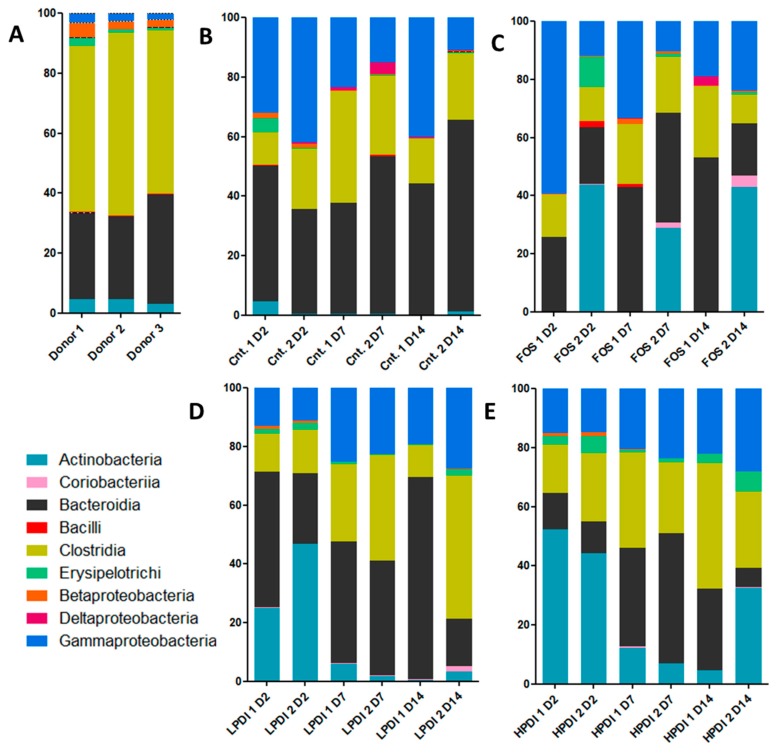
Representation of relative abundance to the class level from the initial time, day 2 (D2), day 7 (D7), and day 14 (D14). Assays were: (**A**) Sample donors; (**B**) Controls (Cnt.); (**C**) Fructooligosaccharides (FOS); (**D**) Low-polymerization-degree inulin (LPDI); and (**E**) High-polymerization-degree inulin (HPDI). Each assay was performed in duplicate.

As expected, the bacterial communities were dominated by the classes Bacilli, Clostridia, and Erysipelotrichia (all three of which belong to the Firmicutes phylum), Bacteroidia (the Bacteroidetes phylum), Actinobacteria, and Coriobacteriia (the Actinobacteria phylum) and Betaproteobacteria, Deltaproteobacteria, and Gammaproteobacteria (Proteobacteria phylum) (Figure 1); Clostridia and Bacteroidia were the two most abundant classes in the inoculum (Figure 1A).

Under all experimental conditions, Gammaproteobacteria were increased, Clostridia were moderately reduced, and Betaproteobacteria were markedly reduced. Coriobacteria, Erysipelotrichia, Bacilli, and Deltaproteobacteria had minor fluctuations over time, except Erysipelotrichia, that were moderately increased with HPDI. Actinobacteria and Bacteroidia continued to be present throughout the fermentation time, but presented considerable fluctuations even among replicates. The control treatment (Macfarlane medium without prebiotic) resulted in the most consistent growth of Bacteroidia, while supplementation with HPDI resulted in the most stable abundance of Clostridia, albeit somewhat reduced compared to the donors’ original microbiotas (Figure 1). 

### 3.2. Alpha-Diversity

**Figure 2:**
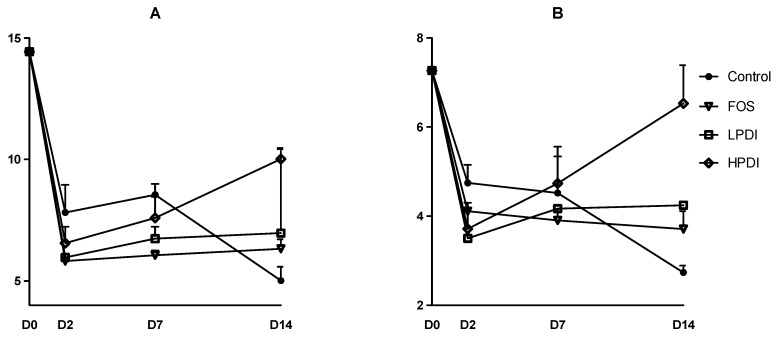
Alpha-diversity at the genus level as per (**A**) the exponential Shannon index (Hill number 1); and (**B**) the inverse Simpson index (Hill number 2).

Two indexes to determine diversity were calculated: the exponential Shannon index and the inverse Simpson index, which correspond to Hill numbers 1 and 2, respectively. Broadly similar trends were observed in both indexes (Figure 2). Diversity was markedly reduced on day 2, but tended to stabilization thereafter for most treatments. At the end of the experiment (day 14), HPDI achieved greater recovery of alpha-diversity, given that it almost reached the initial levels as per the inverse Simpson index. LPDI and FOS had lower alpha-diversity, and the control treatment (no prebiotic) resulted in the lowest alpha-diversity on day 14. A two-way ANOVA analysis with Bonferroni correction indicated that the only significant pairwise difference was that of HPDI against the control on day 14 (*p* < 0.05), while all other pairwise comparisons between groups and days were nonsignificant. 

### 3.3. Stabilization of the System

**Figure 3:**
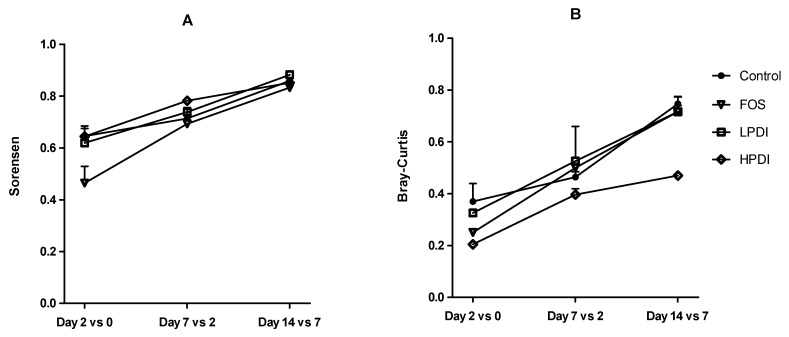
Composition stability, measured as the similarity at the genus level between contiguous sampling days, according to: (**A**) the Sorensen index; and (**B**) the Bray–Curtis index.

Microbiota changes at the genus level over time and within each treatment were studied with the Sorensen and Bray–Curtis beta-diversity indexes. In general, similarities with respect to the previous time point increased over time, thus indicating a more stable composition (Figure 3). The lowest similarity was observed between days 0 and 2, thereby indicating a marked compositional departure from the inoculum. Greater stability was observed for the Sorensen index than for the Bray–Curtis index, especially on days 2 and 7, thus indicating that the more abundant genera played a more significant role in the differences within each treatment over time, as the Bray–Curtis index gives greater weight to these genera compared to the Sorensen index. The similarity between days 7 and 14 was the highest for all treatments, which would suggest that the microbial community composition stabilized, yet a plateau was not observed within the experimental timeframe. A two-way ANOVA analysis with Bonferroni correction indicated that treatment with FOS resulted in the lowest stability on day 2 (i.e., the largest departure from the inoculum), as per the Sorensen index, compared to all other treatments (Bonferroni *p* < 0.05 for the comparison vs control, HPDI and LPDI). Conversely, HPDI had a lower stability than all other treatments on day 14, as per the Bray–Curtis index (Bonferroni *p* < 0.05 for the comparisons vs control, FOS and LPDI), thereby suggesting that the more abundant genera had perhaps not yet stabilized. All other pairwise comparisons between groups and days were nonsignificant. 

### 3.4. Similarity Between Treatments

**Figure 4:**
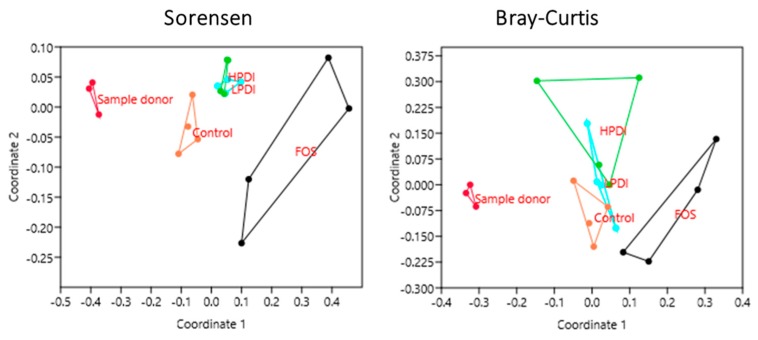
Clustering of samples based on nonmetric multidimensional scaling (NMDS) using the Sorensen and Bray–Curtis indexes at the genus level.

The NMDS analysis revealed a clear closeness between individual donor samples (subsequently pooled as inoculum), but a clear separation between the samples and the microbiotas at days 7 and 14 of ex vivo fermentation, as per both the Sorensen and Bray–Curtis indexes (Figure 4). The Sorensen index clustered together the microbiotas fermented with both the LPDI and HPDI, which were clearly separated from those fermented with FOS or the control medium (no fructan). Conversely, with the Bray–Curtis index, the microbiotas fermented with FOS remained separated, but the LPDI separated further from the HPDI, while becoming closer to the control condition. It is worth noting that sample separation within HPDI in NMDS analysis was markedly larger with the Bray–Curtis index than with the Sorensen one, thus indicating that the variability within HPDI seem to be mostly due to few highly abundant genera.

Microbial composition varied significantly both over time and among treatments, as significant differences were observed upon two-way ANOSIM analysis for the time and treatment factors with both the Sorensen (*p* ≤ 0.0001 for both treatment and time) and Bray–Curtis indexes (*p* = 0.0045 and *p* ≤ 0.0001 for treatment and time, respectively). A pairwise comparison between treatments in the period between days 7 and 14 indicated that the community structure of microbiotas resulting from fermentation with HPDI or LPDI did not differ significantly from one another, while almost all other pairwise comparisons resulted in nominally significant differences. More differences were noted with the Sorensen index than with the Bray–Curtis index, although none of these significances survived the Bonferroni correction (Table 1). Of note, NMDS stress factor was below 0.2 both for Bray–Curtis and Sorensen, indicating that treatment-based clustering was nonrandom. 

### 3.5. pH Variation

**Figure 5:**
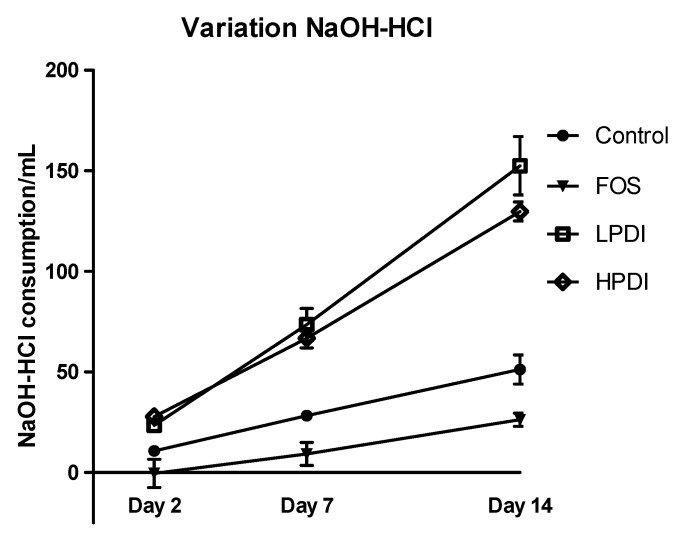
Total acidification activity, expressed as the difference in mL of the consumption of NaOH and HCl required to keep the system’s pH stable, for the different fructans (FOS, LPDI, and HPDI) and the control treatment.

Analysis of the differential amount of NaOH and HCl consumed to maintain the fermenter in the correct pH range (Figure 5) showed that all treatments induced acidification over time (*p* < 0.0001), with significant differences among the treatments (*p* = 0.0012). LPDI and HPDI did not differ from one another, but clearly produced more acidification than either the control or FOS on days 7 and 14 (Bonferroni post-test *p*-value < 0.01 for all pairwise comparisons). Note that acidification with FOS was in fact found to be lower than the control on day 14 (Bonferroni *p* < 0.05). 

## 4. Discussion

The health benefits of prebiotics are widely accepted [46], and the present study provides further evidence that fructan-type prebiotics can modify the human intestinal microbiota. However, fructans with differing degrees of polymerization (FOS, LPDI, and HPDI) had different impacts on the evolution of the microbial community. 

Our study used a simple system to simulate average colonic conditions and test the effects of fructans with different degrees of polymerization in the human gut microbiota. More precisely, our system corresponded to the transverse colon (middle stage), based on a system developed by Macfarlane, with pH and retention time conditions similar to the transverse colon conditions in the SHIME [21]. Conversely, Lacroix [47], with three stages, used a shorter retention time because it simulated the digestive tract of children. The fermenters were inoculated with pooled fecal material from three donors belonging to the same Firmicutes enterotype, which has been reported to be the most common in the population [26]. Moreover, the relative abundances of the microbial communities in the inocula were similar to those described in the literature for healthy patients [8,26]. 

This study showed that all alpha-diversity indexes initially decreased in all treatments. Recent studies that compared the effect of three commercial apple varieties (grated with the skin) versus inulin and cellulose [48], and the effects of FOS, GOS, XOS, and beta-glucan [49], also showed decreased alpha-diversity over time for all treatments. This could be a common effect of ex vivo simulation compared to the real human gut. The results of So et al. 2018 [17] also support the selectivity criteria of the prebiotic concept, in which the host microorganisms selectively utilize the prebiotic as substrates. It could be hypothesized that the loss in diversity is due to fructans stimulating just a few bacterial groups, but our results showed that the control without prebiotic brought about the same behavior, which would rule out fructans as the cause of diversity loss. The metabolization of dietary polysaccharides by the gastrointestinal bacteria is an example of the symbiotic relationship between the host and the microbiota. Furthermore, this relationship provides an avenue for dietary modulation of the microbiota, because microbial growth and metabolism depend on substrate availability, e.g., the type of dietary fiber or prebiotic consumed by the host [20]. The Simpson index (i.e., number of highly effective OTUs) gives more weight to abundant OTUs, compared to the inverse Shannon index (i.e., the number of effective OTUs). Our system showed that diversity decreased with both indexes and, consequently, in both abundant and rare OTUs. Differences among these two indexes showed that fructans with a higher degree of polymerization (HDPI) produced more diversity towards the end of the experiment, and that this effect was statistically significant against controls when more weight was attached to the more abundant (or “highly effective”) OTUs.

Results obtained with our ex vivo fermentation system suggest that the microbiota stabilized from day 7 of fermentation, as the similarity between days 7 and 14 was found to be high, although a plateau was not yet reached or not detected. Macfarlane reported that his system stabilized in 24 h, but only nine bacterial groups where checked with PCR [25]. Conversely, the SHIME takes up to two weeks to stabilize when 130 different bacterial groups are considered [23], a result much more consistent with our findings. Greater stability was observed with the Sorensen index compared to Bray–Curtis index, thus indicating that low-abundance OTUs stabilized faster than highly abundant OTUs, akin to the behavior of alpha-diversity indexes. Moreover, stability after 14 days of highly abundant OTUs was significantly lower for long-chain fructans (HPDI) than for all other treatments. Together with the higher alpha-diversity attained compared to controls, we hypothesize that, given more time, HDPI could produce an even greater increase in alpha diversity.

As expected, differences in the specific microbiota composition were found among treatment groups. However, Bonferroni-corrected statistical significance was not attained in pairwise comparisons, probably owing to the large number of between-group comparisons (six) and the limited number of replicas. 

Taken together, our results show that fructans with a polymerization degree of above 10 (HPDI and LPDI) result in similar microbiota compositions, especially with respect to low-abundant OTUs, and in similarly high acidification activity, compared to the control condition (no fructans). Moreover, the fructan with the highest degree of polymerization (HPDI) restored alpha-diversity to baseline levels compared to the control when more weight was given to highly abundant OTUs (inverse Simpson index). These highly abundant OTUs were *Bifidobacterium, Bacteroides, Prevotella*, *Faecalibacterium*, *Roseburia*, *Acidaminococcus*, *Megasphaera*, *Catenibacterium, Citrobacter*, and three undefined genera belonging to the families *Enterobacteriaceae*, *Lachnospiraceae*, and *Eubacteriaceae*. Our observations also highlight the importance of using different diversity indexes to reveal the differences between more abundant and less abundant OTUs under a given study factor. Moreover, our experiment showed that FOS (polymerization degree 2 to 8) promoted a different microbial composition than longer-chain inulins, and failed to stimulate acidification activity compared to control fermentation without fructans.

Numerous reports link a decreased diversity of the gut microbiota to a declined health status, including conditions such as inflammatory bowel diseases (ulcerative colitis and Crohn’s disease), coeliac disease, allergies, and obesity [50]. Recent simulation studies support a causative role of microbiota diversity in health status [51], and drops in diversity have been associated to a higher risk of subsequent recurrence both in ulcerative colitis [52,53] and Crohn’s disease [54,55]. Previous studies in inflammatory bowel disease have used mixtures of low and medium length inulin-type fructans (i.e., FOS and LDPI) [56]. However, based on our results, HDPI seems a better candidate to promote microbiota diversity to help prevent recurrence in inflammatory bowel disease, and deserves further testing in clinical trials.

One of the limitations of our ex vivo model was our use of a single stage of colonic conditions, corresponding to the transverse colon, rather than multiple stages to simulate the functionality of different locations in the gut. As fibers are normally degraded in the gut, this factor could introduce additional differences not observed in this study. A second limitation of our study was the small number of replicates performed for each treatment, which reduced the statistical power in some analyses. A third limitation was the fact that the effects of the polymerization degree of fructans were tested on a microbiota with the Firmicutes-predominant enterotype, and future studies on other enterotypes are required to compare the results. Any future research should also measure the production of individual short-chain fatty acids in order to gain further information on the effect of the polymerization degree on the metabolic activity of the microbiota, which in our study was limited to total acidification activity. Such research could reveal additional similarities or differences between different fructans. Finally, it would be especially interesting to study the effect of the degree of polymerization when different fructans are combined, or even when other types of fiber such as beta-glucans, polydextrose, acacia gum, pectin, and partially hydrolyzed guar gum are added. The combination of different fibers could be important to avoid feeding a few bacterial groups, and thus promote a more diverse microbiota [20].

## 5. Conclusions

In conclusion, the ex vivo system used in our study allowed us to observe the effect of the degree of polymerization of fructans on the intestinal microbiota. The degree of polymerization of fructans seems to impact their prebiotic effect, resulting in microbiotas with different compositions. Higher polymerization seems to facilitate greater diversity and acidification activity compared to lower polymerization (i.e., FOS), suggesting that inulins of different degree of polymerization produce different effects on the gut microbiota, both at the compositional and metabolic level. Ultimately, adequately powered clinical trials should be conducted to confirm these effects in humans. 

## Figures and Tables

**Table 1:** Significance of the differences between treatments based on pooled data from days 7 and 14, one-way ANOSIM using the Sorensen and Bray–Curtis indexes at the genus level. Reported *p*-values are nominal, and none of them held after the Bonferroni correction was applied. vs.—versus.

Sample	Sorensen Index (*p*-Value)	Bray–Curtis Index (*p*-Value)
Sample Donor vs. Control	0.0261	0.0296
Sample Donor vs. FOS	0.0286	0.0285
Sample Donor vs. LPDI	0.0259	0.0301
Sample Donor vs. HPDI	0.0303	0.0290
Control vs. FOS	0.0274	0.0282
Control vs. LPDI	0.0298	0.1477
Control vs. HPDI	0.0283	0.0284
FOS vs. LPDI	0.0308	0.0283
FOS vs. HPDI	0.0282	0.0818
LPDI vs. HPDI	0.2602	0.1952

## Supplementary Materials

The following are available online at https://www.mdpi.com/2072-6643/11/6/1293/s1, Table S1: Dietary Questionnaire.
