# Supplementary material for: Effect of the Degree of Polymerization of Fructans on Ex Vivo Fermented Human Gut Microbiome
Source: Nutrients. 2019 Jun 7;11(6):1293. doi: 10.3390/nu11061293 (PMC6627432; doi:10.3390/nu11061293)
Supplement: Supplementary file 1 [file nutrients-11-01293-s001.pdf]

Donor	Meal	Three days before donation	Two days before donation	Last day before donation
1	Breakfast<br>(7:20- 7:30h)	2 toast of bread molt with 2 slices turkey ham<br>1 Orange<br>1 Coffee with milk (½ spoonful of brown sugar)		3 toast of bread molt with 3 slices turkey ham
	Second breakfast<br>(11:30-12:00h)	1 apple	1 banana	1 apple
	Lunch<br>(13:30- 13:45h)	Lentils curry (onion, carrot & pepper)<br>Green tea	Zucchini cream (Onion & Potato)	Spinach tortellini with tuna and fried tomato<br>1 tangerine<br>Green tea
	Snack<br>(17:10- 18:00h)	1 apple<br>1 cereal bar with fruit	1 apple<br>1 Coffee with milk with sugar<br>1 candy at 18:30h	1 banana<br>Pineapple juice<br>1 apple<br>Pipe bread
	Dinner<br>(21:30h)	Broccoli & tomato & hard egg<br>1 toast with hummus<br>Yoghurt<br>Chamomile	Salad of canons, tomato, carrots and avocado	Potato omelet with onion<br>Zucchini cream (Onion & Potato)<br>1 apple<br>Chamomile
2	Breakfast<br>(9:45- 10:00h)	Skim milk with oat flakes<br>1 pear	Lemon juice<br>Kefir<br>Apple compote (cinnamon & agave syrup)	
	Lunch<br>(13:00h)	White beans (onion)<br>Tomato & mozzarella<br>1 blood orange	Bomb rice with sauce (tomato, green & red pepper, leek) & hard egg<br>1 banana	White shiro miso soup<br>Mille with pumpkin with carrot & chicken<br>1 banana
	Snack<br>(17:30- 18:00h)	Strawberry yoghurt	Goat kefir<br>Grapes	Rice drink<br>Cookies with honey
	Dinner<br>(21:30- 22:00h)	1 raw carrot<br>Miso & ginger soup<br>2 toast of rye bread with flax seeds, lettuce, tomato & banana	Almonds & raisins<br>Pumpkin, carrot, potato & leek cream<br>2 pancakes corn with goat cheese<br>Backed sweet potato with cinnamon	1 raw carrot<br>Almonds<br>Bomb rice with sauce (tomato, green & red pepper, leek)<br>1 apple

3	Breakfast<br>(9:45- 10:45h)	Coffee with semi-skimmed milk (1 spoonful of brown sugar)<br>2 chocolate cookies	Black tea with sugar<br>2 tangerines	Black tea with sugar<br>4 cookies<br>1 tangerine
	Lunch<br>(13:00- 13:30h)	White beans with clams<br>Yoghurt with sugar	Rice salad with lettuce, tomato, prawns, olives & pickles with mayonnaise<br>1 kiwi	Vegetables cream (celery, leek, carrot & zucchini)<br>2 pieces loin with backed potatoes & onion<br>1 kiwi
	Snack<br>(16:45- 17:45h)	1 tangerine	Yoghurt with sugar	
	Dinner<br>(22:00- 22:30h)	Lettuce salad with surimi, fresh cheese & avocado<br>5-6 cashew nut<br>Slice of bread<br>1 tangerine	Lettuce salad with avocado, fresh cheese and tuna<br>1 mushroom croquette<br>Slice of bread<br>4 cashew nuts	Backed vegetables (artichoke, onion, potato & radicchio<br>2 mushrooms croquettes

Supplementary Table 1. Dietary Questionnaire
